# Comparative proteomic analysis in serum of former uranium miners with and without radon induced squamous lung cancer

**DOI:** 10.1186/s12995-019-0228-y

**Published:** 2019-03-15

**Authors:** Simone Helmig, Günter Lochnit, Joachim Schneider

**Affiliations:** 1 Institute and Outpatient Clinic for Occupational and Social Medicine, Justus-Liebig-University Giessen, Aulweg 129, D-35392, Giessen, Germany; 2 Institute of Biochemistry, Friedrichstraße 24, Justus-Liebig-University Giessen, D-35392, Giessen, Germany

## Abstract

**Summary:**

Former uranium miners of the Wismut Company, East Germany, have been exposed to ionizing radiation from radon decay products and therefore were at high risk for lung cancer. Since histological types of cancer in the so called Wismut cohort revealed an association of high radon exposure with a higher relative frequency of squamous cell carcinoma (SqCC), we used comparative proteomic analysis to identify differentially expressed proteins in serum exposed uranium miners with SqCC.

**Methode:**

Pooled sera of exposed former uranium miners without lung disease and pooled sera of former uranium miners with SqCC were analysed by 2-D gel electrophoresis. MALDI-TOF-MS was performed from reproducable, significantly, at least 5-fold up-regulated protein spots. Proteins were identified by MASCOT peptide mass fingerprint search. Additionally a receiver operating characteristic curve for CYFRA 21-1 was created.

**Results:**

The protein spots were identified as Keratin 10 (K10), Keratin 1 (K1), complement factor H (CFH) and a haptoglobin (Hpt) fragment. The sensitivity for CYFRA 21-1 reveals 60% at a specifity of 95 and 80% at a specifity of 80%. Plotting the sensitivity against specifity reveals an AUC of 0.88.

**Conclusion:**

In SqCC Keratin 10 and 1 were strongly induced. This was associated with CYFRA 21-1, confirming the cytokeratin fragment as a tumormarker.

## Introduction

Uranium minerals are associated with radioactive elements such as radium and radon in the ore which arise from the radioactive decay [1]. Radium decay due to emitted high-LET alpha-radiation has been found to be carcinogenic to humans [2]. Uranium underground miners are primarily exposed to internal ionizing radiation from radon decay products especially α-emitters such as ^222^Rn, ^218^Po, ^214^Pb, ^214^Bi and ^214^Po and also to dust with high contents of crystalline silica via inhalation [3]. To a lesser extent, they are also exposed to uranium ore dust and to external ɣ-rays, both of which are important to consider for risk to organs other than lungs [1]. Uranium mining and processing with a total yield of 231,000 tones of Uranium took place by the Wismut Company, East Germany between the years 1946 and 1990. In this time period, approximately 500,000 to 600,000 employers were exposed to ionizing radiation during underground mining and uranium processing at the Wismut Company. The german federal ministry for the environment enabled the establishment of the so called Wismut cohort with about 59,000 male workers, which is currently worlwide the largest cohort of uranium mine workers (for review refer to [4]. The exposure were in the range from 30 to 300 WLM* per year and about 10–100 mg/m^3^ silica containing dust [3–5]. Especially in the first decade due to unsafe working conditions they were exposed to high radiation levels. With improvement of the working conditions in the later years the avarage exposure level was reduced. More than 20,000 cases of silicosis and more than 9000 cases of lung cancer arised from these working conditions [6]. With an increasing cumulative radon exposure and increasing silica dust exposure a statistically significant increase in mortality from lung cancer has been reported (for review refer to [4]. Even for low cumulative radon exposure a statistically significant association with lung cancer has been shown in Wismut uranium miners [7]. Investigations on the histological types of cancer in the Wismut cohort revealed an association of high radon exposure with a higher relative frequency of smal cell lung carcinoma (SCLC) and squamous cell carcinoma (SqCC) than adenocarcinomas (AC).

Lung carcinomas are frequent occupational cancers in Germany, whereof a great number is caused in Wismut uranium miners by ionizing radiation (so called “Schneeberger” lung cancer) [8]. Cancer is a result of a number of genetic alterations that disturb normal cell growth and differentiation [3]. It is reasonable that different causes of cancer act via different biological mechanisms that reflect the histoplogical subtypes. A pilot study investigating biomarkers with regard to lung cancer has been implemented earlier [9]. In the past decades, the investigating methods and possibilities have been envolving rapidly. Since histological types of cancer in the Wismut cohort revealed an association of high radon exposure with a higher relative frequency of squamous cell carcinoma (SqCC) we used modern technology i.e. comparative proteomic analysis to screen this cohort and to identify differentially expressed proteins in serum of former uranium miners without lung disease and uranium miners with SqCC. Comparative proteomic analysis may be able to identify differentially expressed proteins in serum of former uranium miners without lung disease and uranium miners with SqCC.

## Materials and methods

### Subjects

Within the framework of a former multi-center, molecular biology-based project, *n* = 106 former uranium miners of SDAG Wismut (66.6 ± 4.5 years; 15% current smokers, 80% ever smokers) without lung disease and *n* = 21 former uranium miners of SDAG Wismut (67.5 ± 5.8 years; 57% current smokers, 90% ever smokers) with lung cancer related to ionizing radiation (radon and its decay products) with accepted occupational disease (No. 2402 BKV) (criteria for diagnosis: see [10]) were included, (for detailed description refer to [3]. The histological classification of the lung cancers involves 15 squamous carcinomas, 2 adenocarcinomas, 2 small-cell carcinomas and 2 large-cell carcinomas. For the proteomic analysis within this study, only sera of highly exposed, former uranium miners without lung disease and those suffering from squamous cell carcinomas related to ionizing radiation (radon and its decay products) were examined. This is due to the small number of cancers other histological classification not suitable for proteomics discrimination and further statstical analysis. Former uranium miners of SDAG Wismut without lung disease are indicated as “former uranium miners without lung diesease” and former uranium miners of SDAG Wismut with sqouamous lung cancer are indicated as “uranium miners with SqCC”.

The miners had started their work within the Wismut Company between 1946 and 1978, and over 50% had stopped working as miners before 1980. The mean time of employment at the company was 21.8 years (range: 0.4 bis 42.1 years). Work in the underground mine was for about 14.5 years, and exposure to ionising radiation in uranium processing was calculated at 7.4 years. Cumulative exposure in WLM* was calculated in each UM by ZeBWis (special department of the German Professional Associations for the observation of the health of former Wismut workers). The mean exposure was calculated at 612 ± 500 WLMs (range: 0,7–1954 WLM) or 6.4 ± 9.76 kBqh/m^3^ (range: 0.06–80 kBqh/m^3^) and 53.5 ± 101.7 mSv (range: 0–874.3 mSv) respectively.

All sera were frozen following venipuncture and centrifugation, and stored in liquid nitrogen until analysis.

### Sample preparation

Serum samples were clarified by centrifugation (20,500×g for 20 min at 10 °C). One milliliter of the supernatant was applied to the Proteominer Enrichment Kit (large capacity kit, BioRAD) according to the manufacturer’s instructions.

### Two-dimensional gel electrophoresis

Detailed descriptions of laboratory methods and statsitical analysis are presented in Korfei et al. 2011 [11]. In brief: For 2-D gel electrophoresis proteins solubilized in 6 M urea, 2 M thiourea, 4% CHAPS, 1% DTT and 2% Pharmalyte 3–10. IPG-strips (pH 3–10 nl) were rehydrated at 20 °C with the protein extract. On each strip, 63 or 75 μg proteins were applied and isoelectric focusing was performed with 32.05 kVh. After focusing, the IPG-strips were equilibrated for 10 min in 2 ml equilibration stock solution (ESS; 6 M urea, 0.1 mM EDTA, 0.01% bromphenol blue, 50 mM Tris-HCl pH 6.8, 30% glycerol) for 15 min in 2 ml ESS I (10 ml ESS containing 200 mg SDS, 100 mg DTT) followed by 15 min in ESS II (10 ml ESS containing 200 mg SDS, 480 mg iodacetamide). Protein separation in the second dimension was performed by electrophoresis on 12.5% SDS polyacrylamide gels according to Laemmli (Laemmli, 1970). Electrophoresis was carried out at least in duplicat using a Hoefer 600 system with the following program: 15 min at 15 mA/gel and 5 h at 110 mA at 25 °C. Gels were stained with Flamingo (BioRAD) and scanned with a Typhoon 9100 (GE Healthcare). Densitometric analysis of the gels was done with PDQuest (BioRAD). Protein spots present in all gels and showing statistical significant differences in abundance (five fold up-regulated) were selected for further analysis.

### Tryptic in-gel digestion of proteins

Stringent criteria such as spots must be present in all gels, spots must be at least five fold upregulated with at least 98% confidence interval were applied to select spots. Selected spots were digested after reduction and carbamidomethylation with trypsin using an automated liquid handling system (MicroStarlet, HamiltonRobotics, Martinsried, Germany). Tryptic peptides were eluted from the gel plugs with 1% trifluoric acid.

### Matrix-assisted laser-desorption ionization time-of-flight mass spectrometry (MALDI-TOF-MS)

MALDI-TOF-MS was performed on an Ultraflex TOF/TOF mass spectrometer equipped with a nitrogen laser and a LIFT-MS/MS facility. The instrument was operated in the positive-ion reflectron mode using 2,5-dihydroxybenzoic acid and methylendiphosphonic acid as matrix. Sum spectra consisting of 200–400 single spectra were acquired. For data processing and instrument control the Compass 1.4 software package consisting of FlexControl 3.4, FlexAnalysis 3.4 and BioTools 3.2 was used.

### Database search

Proteins were identified by MASCOT peptide mass fingerprint search (http://www.matrixscience.com) using the NCBInr database (20,140,811; 47,570,513 sequences; 16,962,606,718 residues; 247,733 human sequences) and Uniprot_human (20,151,014; 92,006 sequences; 36,592,947 residues). For the search a mass tolerance of 75 ppm was allowed and carbamidomethylation of cysteine as global modification and oxidation of methionine as variable modification were used. A false positive rate of 5% was allowed.

### CYFRA 21-1

Sera were kept frozen at − 18 °C until analysis was carried out. CYFRA 21-1 analyses were performed in sera using reagents from Roche Diagnostics^®^ GmbH, Mannheim, Germany, and were measured with an ES^®^ 600 ELISA analyzer (Roche^®^, Mannheim, Germany).

## Results and discussion

It is noteworthy that both comparison groups i.e. former uranium miners without lung disease and uranium miners with SqCC had significant occupational radiation exposures, which alleviates a potential bias, which would arise for the case with a control group without radiation exposure.

**Fig. 1:**
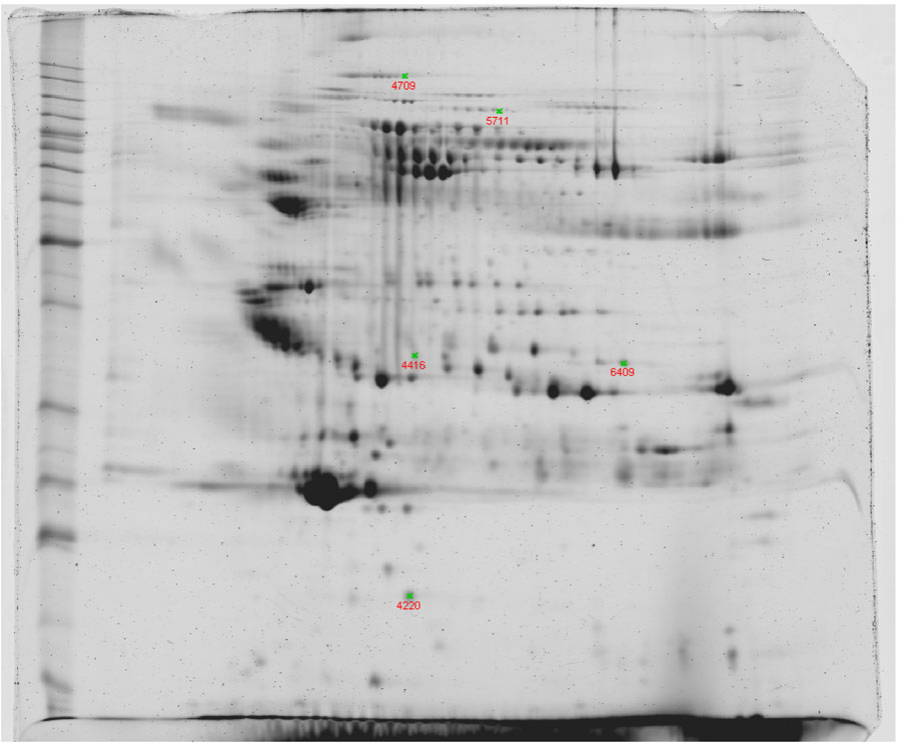
Representative 2-DE map of 75 μg of protein extracted from sera of uranium miners with SqCC. Separation of extracted protein was performed on linear strip with a pH range of 3–10 followed by 12,5 SDS-PAGE. Gels were stained with FLAMINGO (BioRad). 2D gels were scanned and differences in protein abundance were detected using Proteomweaver software. Proteins that differed more than or equal to 5-fold and were identified are marked

A full comparison of all protein spot visualized in three different proteome maps of sera from exposed healthy uranium miners and sera of uranium miners with SqCC was performed. In order to increase the specifity rather stringent criteria (present in all gels and at least five fold upregulated with with at least 98% confidence interval were applied. This approach revealed 5 protein spots which fullfilled the aforementioned criteria, while protein spots with a lower specifity were not considered within this study. The 5 protein spots could be identified by MALDI-TOF-MS after in-gel digestion (Table 1**)**. A representative 2-D gel is shown in Fig. 1. The upregulated protein spots were identified as Keratin 10 (K10), Keratin 1 (K1), complement factor H (CFH) and a haptoglobin (Hpt) fragment. Information about the identification of these differentially expressed protein spots is provided in Table 2.

**Table 1:**
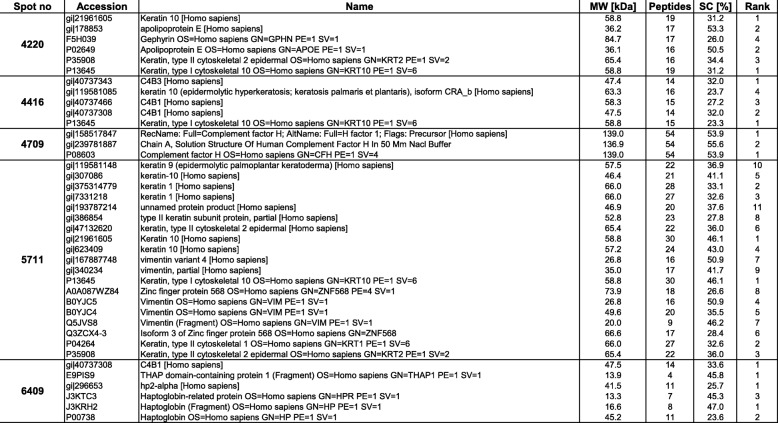
Database screening of MALDI-TOF-Analysis

**Table 2:**
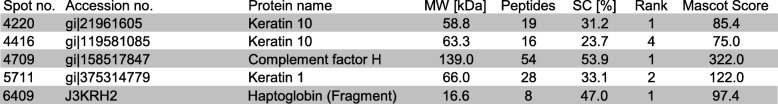
Proteins significantly up-regulated in SqCC relative to healthy controls

**Fig. 2:**
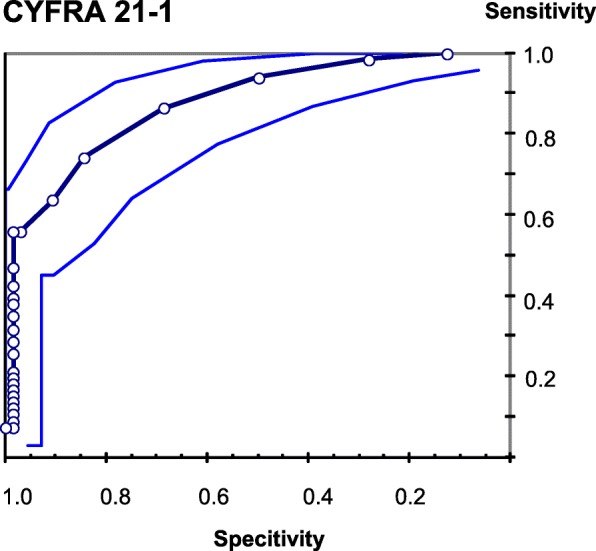
ROC of CYFRA 21-1. Receiver operating characteristic curves (ROC) and confidence intervals comparing the performance of CYFRA 21-1-measurements in sera of 108 healthy uranium miners and in sera of 18 uranium miners with squamous cell carcinoma. AUC (Area under curve) = 0.88

A very important aspect of this study is the evidence of increased keratins K1 and K10 in the serum of uranium miners with SqCC. Keratins are intermediate-filament-forming proteins, which are primarily expressed in epithelial cells [12]. Human keratins represent the type I keratins K9–K10, K12–K28, and K31–K40 and the type II keratins K1–K8 and K71–K86 [13]. Since tumors usually retain the keratin patterns of their epithelial origin, their keratin patterns are being used for cell and tumor typing. Additionally, keratins are often used as epithelial differentiation and tumor markers [13]. Keratin expression of K1 and K10 is assigned especially to the postmitotic suprabasal epidermal cells, wereas the proliferative basal cell layers show different expression patterns (K5, K14, K15) [13]. Besides maintaining the mechanical integrity, K10 also inhibits proliferation and cell cycle progression of keratinocytes [14, 15]. The loss of K10 results in a increased keratinocyte turnover (for review, see [16]. K1 and K10 seem to be focally expressed in suprabasal cells of internal noncornifying stratified squamous epithelia. Especially in relation to maturation and keratiniziation focal expression of K1 and K10 can be seen in squamous cell carcinomas of internal organs and skin (for reference, see [17]. K1 and K10 so far have not been applied for tumor diagnostic but can be regarded as “keratinozation markers” of keratinocytes [13]. Using commercially available ELISA kits did not achieve usable results for the detection of K1, K10 and Vimetin in sera of exposed uranium miners. Investigating sera and controls by western blot analysis using different antibodies (i.a. Fa. Abcam) revealed varying results in dependence of the applied antibody. Therefore these results were not applicable. Soluble keratin fragments released by carcinoma cells (mainly K8, K18, and K19 fragments) in the serum of cancer patients are used to monitor disease progression in the case of certain carcinomas such as non-small cell lung cancer (NSCLC) [18, 19]. For example increased concentrations of CYFRA 21-1, a water-soluble K19 fragment, are present in tumors of squamous origin, including the lung [20]. The highest sensitivity, between 40% [21] and 100% [22] was reported for SqCC, whereby the sensitivity rises with the progression of the disease [20]. Not only the diagnostic but also the predictive and prognostic value of CYFRA 21-1 for advanced NSCLC has been reported [23]. Within the previously described cohort of former uranium miners of SDAG Wismut a receiver operating characteristic curve for CYFRA 21-1 was created (see Fig. 2). The sensitivity for CYFRA 21-1 in the uranium miners suffering from squamous cell carcinomas related to ionizing radiation reveals 60% at a specificity of 95 and 80% at a specificity of 80%. Plotting the true positive rate (sensitivity) against the true negative rate (specificity) reveals an area under the curve (AUC) of 0.88. Therefore CYFRA 21-1 confirmed as a serum biomarker in SqCC.

Further markers such as the tumor suppressor gene product p53, the epidermal growth factor receptor (EGF-R) or Anti-p53 antibodies could not be identified within this study and in concordance with former results are not useful as serum biomarkers for detection of lung cancers due to ionizing radiation [3].

The complement system is a cascade of functionally related proteins, which mediate the immune respose leading in consequence to cell lysis. The activation of this system is highly controlled by several regulatory proteins. Human complement factor H (CFH), is a member of the regulators of complement activation family and plays a central role in the inhibition of the activation of the alternative pathway [24, 25]. Especially human NSCLC cells have the ability to circumvent the complement mediated immune response and are resistant to complement mediated cell lysis [26]. There is some evidence suggesting the involvement of CFH in carcinogenesis by demonstrating a protective role for CFH. For example a high expression of CFH was shown in most non-small cell lung cancer cell lines with a protective effect against complement activation [24]. Evidence for a role of CFH in squamous cell cancer of skin (cSCC) progression has been provided and CFH has been suggested as progression marker and potential therapeutic target in cSCC [27]. CFH has also been suggested as a diagnosic marker for lung adenocarcinoma and transitional cell cancer of the bladder [25, 28]. The use of monoclonal antibody proteomics of a panel of biomarkers, showed statistically significant differences for five biomarkers including CFH and haptoglobulin (Hpt) in the plasma of NSCLC patients (*N* = 301) vs. healthy controls (*N* = 235) [29]. Examination of the complement system is clinically relevant in various immune complex diseases, such as systemic lupus erythematosus, vascular inflammations, glomerulonephritis or angioneurotic edema. Therefore the CFH is rather not a specific diagnostic maker for lung cancer diseases.

The comparison of serum from patients with a newly diagnosed NSCLC to serum from healthy controls, by using 2-dimensional difference gel electrophoresis (2D-DIGE), identified haptoglobin (Hp) and glycan-modified derivatives as differentially expressed proteins. Therefore haptoglobin (Hp) and glycan-modified derivatives were suggested to be potentially useful markers for the clinical diagnosis of NSCLC [30]. Comparing serum proteomic profiles of 11 SCC patients, 7 chronic obstructive pulmonary disease (COPD) patients and 7 healthy smokers as controls identified the HP peptide HP216 as a promising cancer biomarker for the early detection of SCC in high risk lung cancer populations, including COPD patients and heavy smokers [31]. Using 2-D liquid phase fractionation system (PF2D) and mass spectrometry Hp reached a sensitivity of 89% and an AUC of 0.929 for lung adenocarcinomas when the cohort was restricted to male subjects [32]. Hp is an acute phase protein that scavenges haemoglobin released into circulation for example during haemolitic processes. The plasma concentration increases clearly during inflammation such as infections, injury or malignancy [33]. IL-6 is an major inducer of this protein [34]. Therefore, an increased Hpt serum concentration is an unspecific marker for inflammation, wereas a decreased Hp serum concentration is an marker for haemolytic processes.

## Conclusion

In this study the evidence of increased keratins K1 and K10 in the serum of uranium miners with radiation-induced SqCC is notable. K10 and K1 may be valuable for tumor diagnosis in sera. As a surrogat cytokeratin fragment CYFRA 21-1 proofed to be an established serum biomarker for SqCC. The upregulated proteins CFH and Hpt are rather non-specific diagnostic makers for lung cancer diseases.
